# The New Zealand Asthma and Allergy Cohort Study (NZA^2^CS): Assembly, Demographics and Investigations

**DOI:** 10.1186/1471-2458-7-26

**Published:** 2007-02-28

**Authors:** Michael J Epton, George I Town, Tristram Ingham, Kristin Wickens, David Fishwick, Julian Crane, the New Zealand Asthma and Allergy Cohort Study Group

**Affiliations:** 1 Canterbury Respiratory Research Group, Department of Medicine, Christchurch School of Medicine and Health Sciences, University of Otago, PO Box 4345, Christchurch, New Zealand; 2 Wellington Asthma Research Group, Department of Medicine, Wellington School of Medicine and Health Sciences, University of Otago, Wellington, New Zealand; 3 Centre for Workplace Health, University of Sheffield, Sheffield, UK

## Abstract

**Background:**

Asthma and allergy are highly prevalent in industrialised countries. Longitudinal and cross-sectional studies have identified a number of potential risk factors for these conditions, including genetic and environmental factors, with significant gene-environment relationships. Birth cohort studies have been proposed as an important tool to explore these risk factors, particularly exposures in early life that are associated with later disease or protection from disease. This paper describes the establishment of a birth cohort in New Zealand.

**Methods:**

A birth cohort was established in 1996 in Christchurch and Wellington and infants recruited between 1997–2001. Expectant mothers were recruited by midwives. Children and mothers have undergone assessment by serial questionnaires, environmental assessment including mould and allergen exposure, skin-prick testing, and at age six years are undergoing full assessment for the presence of asthma, atopy and allergic disease, including genetic assessment.

**Results:**

A total of 1105 children have been recruited, and the retention rate at fifteen months was 91.4%. 15.2% of the children at recruitment have been identified as Maori. A positive family history of asthma, eczema or hay fever has been reported in 84% of children. All children have now been assessed at fifteen months and 685 children from the cohort have reached age six years and have completed the six year assessment.

**Conclusion:**

The cohort is fully assembled, and assessment of children is well advanced, with good retention rates. The study is well placed to address many current hypotheses about the risk factors for allergic disease and asthma.

## Background

The incidence of atopy, allergic disease and asthma is increasing in industrialised nations, including New Zealand. Longitudinal birth cohorts such as in Tucson USA [1] Melbourne [2] and Perth [3] Australia, and the German multi-centre atopy study (MAS-90) [4], as well as cross-sectional studies such as Belmont [5] in Australia have identified a number of potential risk factors for the development of atopy, allergic disease and asthma. These include a genetic predisposition, foetal growth [6] and a variety of early life environmental factors.

Exposure to house dust mite allergens is strongly associated with sensitisation [7], and in turn sensitisation is strongly associated with asthma [8]. The relationships between allergen exposure and the development of asthma appear more complex [9]. New Zealand has amongst the highest levels of house dust mite allergen in the world, and is therefore well placed to further explore such relationships. Although a birth cohort established 34 years ago in Dunedin has reported on determinants of asthma and atopy [8,10], early life exposure information from this cohort is sparse, preventing detailed examination of current hypotheses.

This paper describes the establishment of a New Zealand birth cohort, to explore environmental and genetic factors associated with the development of atopy, allergic disease and asthma. Specifically we wished to explore the influence of perinatal factors, anthropometric measurements, infant infections, family size, immunisation, diet, environmental allergen and environmental tobacco smoke exposure on the development of atopy, allergic disease and asthma. There are a number of reasons for mounting such a study in New Zealand, including high prevalence of allergic disease and asthma, high allergen levels, and low industrial pollution levels. In addition, the New Zealand population has been extensively studied in the context of other international work [11,12], allowing comparisons with previous studies to be undertaken. There is growing recognition of the importance of longitudinal birth studies in trying to understand the true progression of asthma and atopic disease and to identify risk factors [13]. Equally it is important to recognise their limitations and to document the assembly and investigation of birth cohorts. This has also recently been recognised by the publication of the study designs of asthma and allergic disease birth cohorts in Europe [14].

In this paper we have therefore summarised the assembly and study of a birth cohort of infants in New Zealand to age eight years.

## Methods

A prospective birth cohort of 1000 infants born in two metropolitan centres (Christchurch, population 417,000, and Wellington, population 420,000) in New Zealand was established in 1997. Planning for the study started in 1996. The two centres were chosen because there were established respiratory research organisations in both cities, and the requirements for large numbers of infants and potential recruitment challenges necessitated two-centre recruitment. Details of the timeline for all assessments are given in Table 1.

**Table 1:** Investigation Timeline

	**Recruitment/Birth**	**3 Months**	**6 Months**	**15 Months**	**2 Years**	**3 Years**	**4 Years**	**5 Years**	**6 Years**	**8 Years**
**QUESTIONNAIRE**										
Birth Details/Parity	x									
Diet/Feeding Practices	x	x	x	x					x	
Family Health										
-Parental Health		x							x	x
-Siblings Health		x		x	x	x	x	x	x	x
Outcome Measures										
-Asthma & Atopy		x		x	x	x	x	x	x	x
-Medication Use		x		x	x	x	x	x	x	x
-Healthcare Contacts		x		x	x	x	x	x	x	x
-Infections/Illnesses		x		x	x	x	x	x	x	
Allergen Avoidance									x	x
Vaccinations		x		x	x	x	x	x	x	x
Indoor Environment		x		x	x	x	x	x	x	x
Smoking		x		x			x	x	x	x
Childcare				x	x	x				
Exercise & Activity									x	
Indoor Swimming Pools									x	
										
**OBJECTIVE MEASURES**										
Anthropometry	x			x					x	x
Cord Blood Collection	x									
Dust Collection										
-Childs bedroom floor		x		x						
-Childs bed		x		x						
Skin Prick Testing				x					x	
Spirometry									x	
Exhaled Nitric Oxide									x	
Nitrogen Dioxide (home)									x	
Hair Nicotine Sample									x	
Blood Collection									x	
										
**MOTHERS**										
Diet		x		x						
Anthropometry				x						

Mothers and infants were recruited through midwives between 1997–2000 in Wellington and 1998–2001 in Christchurch. Expectant mothers were approached by their midwives to give written informed consent to be enrolled in the study prior to delivery. Midwives were selected randomly from all midwives practising in Wellington and Christchurch and were trained and supported by trial coordinators. Midwives were encouraged to discuss the study and try and enrol all expectant mothers under their care, to reduce selection bias. No explicit attempt was made to recruit families with a history of atopy, allergic disease or asthma, indeed the importance of enrolling all mothers was stressed. There were no explicit exclusion criteria in the peri-natal period; in particular non-singleton pregnancies were able to be included. Midwives were asked to keep a record of all mothers that they approached and to collect basic demographic information on them in particular mothers who declined to participate. In addition, efforts were made to ensure Maori participation by ongoing contact with Maori health providers and consumer groups. All mothers gave written informed consent for their children to participate in this study to monitor their health and development and undergo regular assessments in the form of interviews, questionnaires and a variety of specific tests. The initial informed consent process covered assessments for the first five years, with subsequent consent for the six and eight year assessments being obtained prior to these assessments (including consent for DNA collection, storage and genetic analysis-see below). Verbal agreement was obtained from each child prior to the six and eight year assessments, as required by our local ethics committees; if a child refused any particular segment, this was not undertaken.

All assessments were administered by trained research staff, using a pre-agreed manual of procedures. Extensive pre-testing of assessment procedures undertaken on non-participant families prior to use, and ongoing inter-centre quality control of assessments and staff was undertaken through the study.

Ethics committee approval for the conduct of the study was obtained from the Wellington and Canterbury Regional Ethics Committees.

In 1993 and 1994 a pilot study of 154 new born infants was undertaken in Wellington [15]. These children were followed with visits at 15 months and 6 years. This pilot provided insights into the design and practical problems associated with large cohort studies, in particular problems with the skin prick testing. There was an initial response rate of over 90% with a 15 months follow-up rate of 97%.

The statistical rationale behind a cohort of 1000 children was based on this pilot data for the major risk factors, and the prevalence of asthma was based on New Zealand research available at the time. At 15 months, with a follow-up rate of 90%, a prevalence of atopy of 20%, a prevalence of the major risk factors of 20% (e.g. high house dust mite exposure) the study will have 95% power to detect a relative risk of atopy of 1.7. At 6 years, with a follow-up rate of 75%, a 12 month period prevalence of asthma symptoms or atopy of 20%, a prevalence of the major risk factors of 20%, the study will have >90% power to detect a relative risk of 1.7. These assumptions are conservative and are in keeping with the findings of the ISAAC study, which found the prevalence of asthma symptoms in six-to seven-year old children to be 25% [11]. Furthermore our follow-up rate at 6 years is greater than the 75% predicted.

## Assessments and data acquisition

### Questionnaires

All questionnaires have been informed by previous New Zealand studies [8,11], together with new questions addressing specific hypotheses.

### Recruitment visit

Expectant mothers were recruited into the study by participating midwives and consent for participation was confirmed soon after birth by the study team.

### Birth

At birth, midwives or study nurses collected data about the birth including any complications. Anthropometric details were collected including birth weight, length and head circumference. Cord blood was collected by the midwife for measurement of IgE.

A face to face questionnaire interview was administered including demographic details, initial feeding practices, delivery details, infant's health status, and medications (including Vitamin K).

### Three month home visit

A face to face questionnaire was administered to collect information about ethnicity (parental-identification of child's ethnicity as recommended by the Population Census in New Zealand), family details, occupation and socio-economic status, breast- and/or formula-feeding practices, introduction of solid foods, and diet, child's health and vaccinations, medications, indoor environment, smoking, and child care.

Indoor dust samples were taken from child's bedding and bedroom floor for measurement of the house dust mite and cat allergens Der p 1 and Fel d 1, endotoxin, peptidoglycans and beta glucans as a marker of fungal biomass. A Hitachi CV-2500 vacuum cleaner (1100 W) was used for vacuuming, with the dust collected using a 25 μm pore nylon mesh sock. In the bed, an area of 0.25 square meters was vacuumed for two minutes, on the item that was immediately below the blanket/sheet the child slept on. On the bedroom floor, an area of one square meter was vacuumed for one minute on carpet or rugs, or for two minutes on bare floorboards. The samples were labelled and placed in plastic bags, and then stored at -20°C until they were sifted and analysed. Der p 1 and Fel d 1 were analysed by ELISA [16,17]. Endotoxin was measured by kinetic chromogenic Limulus Amoebocyte Lysate (LAL) method (Bio Whittaker, Walkersville, MD, USA). A measure of peptidoglycan exposure was determined by quantifying muramic acid levels present in house-dust samples using ion-trap gas chromatography-tandem mass spectrometry (GC-MS-MS) [18]. Beta glucans will be quantified using ELISA [19].

The child's mother reported the presence of damp and mould, including a damp or musty smell in the whole house. This data was also collected for the child's bedroom by the interviewer [20].

### Six month telephone questionnaire

A questionnaire to collect information about feeding practices, introduction of solid foods, and diet.

### Fifteen month home visit

A comprehensive health questionnaire was administered as at three months including a full dietary questionnaire covering breast- and/or formula-feeding practices over the previous twelve months, current feeding practice, general eating pattern and the consumption frequency of selected foods.

Anthropometric measurements were taken including height, weight, head circumference, and upper arm circumference. Maternal height and weight was also measured.

Skin prick tests were performed using the following allergens; *Dermatophagoides pteronyssinus*, Cat, Dog, Rye Grass, Cow's milk, Egg white, Peanut, Aspergillus, Cockroach, Histamine, Negative control. We used the Quintest™ system (Bayer Corporation, West Haven, CT) for skin prick testing [21], having found difficulty in using the standard methodology in 15 month old infants during the pilot. The Quintest has five lancets per device which allows for quick administration of the test. The devices were dipped in a tray carrying ten of the allergens and then applied to the child's back in quick succession. Two Quintest devices were used and one test (dog) was done separately using a single lancet. After fifteen minutes, wheals were measured using standard techniques and the average of the perpendicular diameters calculated. A positive allergen response will be defined as an allergen to histamine ratio of > 0.5, after subtraction of the negative control, and atopy defined as at least one positive response [22].

Further collections of dust from bed and bedroom floor were undertaken (see above), allowing comparisons of allergen and endotoxin levels between three and fifteen month assessments.

### Two, three, four and five-year telephone questionnaires

Telephone questionnaires were subsequently undertaken at two, three, four and five years. These questionnaires covered family health, respiratory problems, health service utilisation, dietary intake, vaccinations, indoor environment, and childcare. In addition, in the four- and five-year questionnaires we included questions updating environmental tobacco smoke exposure, types of bedding used by children, exposure to pets, and paracetamol use.

### Six-year assessment

The extensive six-year assessment is currently being undertaken on children in the cohort at the Wellington and Christchurch research centres and is focussed on a detailed enquiry about risk factors and obtaining a number of objective markers of asthma and atopy. This assessment includes the following investigations:

### Questionnaire

The questionnaire, which has questions on outcome measures largely taken from the ISAAC Phase II questionnaire, is designed for completion by the parent of the child. It includes questions about the following: the occurrence and severity of respiratory and atopic symptoms; presence of asthma and atopy; infections; healthcare contacts; medication usage (including paracetamol and antibiotics); vaccination history; swimming pool visits; diet and physical activity; family size; family history of atopic diseases; and the indoor environment including: environmental tobacco smoke exposure (with additional questions exploring duration of exposure from birth), bedding, pets, crowding, dampness and mould, cooking and heating fuels.

### Anthropometry

Anthropometry protocols are based on the Australian Food and Nutrition Monitoring Unit (AFNMU) Expert Panel Guidelines (2001) adapted from the measurement protocol described by the World Health Organisation (WHO Expert Committee 1995). Height is assessed using a stadiometer (Wellington: GPM, SibnerHegner, Zurich, Switzerland; Christchurch: KaWe Medizintechnik, Asperg, Germany). Weight is assessed using Tanita HD-316 digital scales (Tanita, Tokyo, Japan).

### Exhaled Nitric Oxide

This is being measured, using a Niox^® ^chemiluminescence analyser (Aerocrine AB, Stockholm, Sweden), to ATS standards [23] prior to the child undertaking any forced expiratory manoeuvres. Quality control protocols and reproducibility testing between investigators ensure valid and acceptable results.

### Spirometry

Resting spirometry is being undertaken to ATS standards [24], using a Vitalograph Pneumotrac 6800 Fleisch-type Pneumotachograph and Spirotrac IV software (version 4.20) (Vitalograph, Buckingham, England). Bronchodilator reversibility is also being undertaken on all children fifteen minutes following the administration of inhaled salbutamol 200 μg using a Volumatic™ spacer (Allen and Hanburys Ltd, UK). All spirometry is being undertaken by trained research staff in research laboratories. Within centre calibration biological control programmes ensure technically acceptable and reproducible spirometry.

### Skin Prick Testing

Skin prick testing is being undertaken to the following allergens (Dome/Hollister-Stier, Spokane, WA): *Dermatophagoides pteronyssinus*, perennial rye grass, olive tree (*Olea europaea*), cat pelt, dog hair (dander), horse hair, cockroach mix (american, german), egg white, peanut mix (runner, virginia, spanish), cow's milk (whole), *Aspergillus fumigatus *and *Alternaria tenuiis*. Positive control (Histamine 10%) and negative control are also performed.

Because of problems associated with the Quintest (See Discussion) we adopted a more traditional methodology with allergens being applied to the volar aspects of both forearms with a wooden applicator. Vertical punctures are then made using individual stainless-steel lancets (Dome/Hollister-Stier, Spokane, WA). Positive control is measured at ten minutes, and all other wheals measured at fifteen minutes [25], with wheals traced using a fine-tip pen. A permanent record is transferred to the results booklet using Micropore™ tape (3 M Healthcare, Saint Paul, MN). Mean wheal diameter is measured using both the longest and orthogonal diameters, recorded to the nearest 0.5 mm. A positive reaction is defined as a mean wheal diameter of 3 mm or greater to any allergen, with atopy defined as any positive reaction.

Observer internal-consistency and inter-observer variability are both monitored within and between centres. Coefficients of variation in histamine wheal size for each investigator are required to be less than 0.2; this is tracked throughout the study and addressed with retraining where disparate.

Children are asked to refrain from medications and caffeine-containing drinks according to a protocol sent to the mothers prior to the visit. The only exception is antihistamines where we ask the child to return if there is no response to histamine on skin prick testing. The timing of all medications taken prior to the visit is recorded.

### Assessment of Atopic Dermatitis

This is being undertaken using the UK Atopic Dermatitis Diagnostic Criteria Working Party protocol [26], including a series of standardised questions and visual inspection for the presence of visible flexural dermatitis. The examination involves inspection of the skin around the eyes, around the sides and front of the neck, in front of the elbows, behind the knees, and in front of the ankles. The presence or absence of signs of visible flexural dermatitis is recorded for each of the five areas. Standardisation is ensured through the incorporated training certification process, which includes a photographic observer's manual containing high-quality clinical photographs.

### Blood Samples

Blood samples are being collected by venepuncture, with topical anaesthesia applied prior to venepuncture in all children.

10 mL of serum is frozen and stored initially at -20°C, and then transferred into -80°C for longer-term storage. Serum will be analysed for total IgE levels and allergen-specific antibody levels using CAP FEIA (Pharmacia Diagnostics, Uppsala, Sweden). Further aliquots are being analysed for strain-specific antibodies against currently circulating strains of Respiratory Syncytial Virus (RSV), subtypes A and B by an enzyme linked immunosorbent assay (ELISA).

12 mL of whole blood is also collected as part of the same venepuncture into EDTA sample tubes for Selenium analysis and DNA extraction.

### Selenium Analysis

4 mL of whole blood is frozen immediately after venepuncture to -20°C for glutathione peroxidase (GSHPx) analysis. A further 4 mL, for selenium analysis, is centrifuged (2,000 revolutions per minute for 10 min) and aliquoted into a 2 mL microcentrifuge tube. This plasma is then immediately frozen after separation.

Plasma selenium is determined by graphite furnace atomic absorption spectrophotometry, using a modified version of the method of Jacobson and Lockitch [27]. Samples are analysed in duplicate using a Perkin-Elmer Model 3100 atomic absorption spectrometer (Perkin-Elmer Corp., 761 Main Avenue, Norwalk, Connecticut 06859-0001).

Whole blood GSHPx is analysed using a Cobas Fara autoanalyser with a modified version [28] of the method of Paglia and Valentine [29]. A stock sample of whole blood is analysed during each GSHPx run, as an internal biological control.

### DNA Extraction

The remaining 4 mL EDTA tube is immediately chilled to 4°C for subsequent DNA extraction and storage. In addition to the blood sample for DNA extraction, a buccal DNA specimen is being collected as a contingency, or in the event that venepuncture is not possible. This is collected using an Endoscan plus cytology brush by brushing the buccal mucosa for 15 seconds each side. The tip of the brush is then cut and placed into a 2 mL cryovial containing 50 mM genomic quality sodium hydroxide (0.1 g in 50 mL molecularly pure water). This is then immediately vortexed for 20 seconds and the brush tip discarded. The specimen is then immediately frozen initially to -20°C then transferred for longer-term storage at -80°C. Genomic DNA will be extracted from buccal samples using the method described by Richards and co-workers [30].

### Hair Nicotine

A hair sample (2–5 mg) is being collected and will be assayed for nicotine to objectively assess personal exposure to environmental tobacco smoke, using reversed-phase high-performance liquid chromatography (HPLC) with electrochemical detection [31].

### Nitrogen Dioxide exposure

There is growing concern in New Zealand about the health problems associated with poor housing, in particular cold and dampness. We have shown that 30% of New Zealand households use unflued bottled gas heaters to heat their homes [32]. These heaters are a significant source of nitrogen dioxide, which has been associated with increased asthma morbidity. A Nitrogen Dioxide passive diffusion (Palmes) tube (Gradko International Ltd, Hampshire, UK) is placed in a standardised location in each child's home for 14 days following the child's assessment [33].

### Eight-year questionnaire

Largely similar to the six-year questionnaire this will collect information on the major outcome measures and key risk factors, including indoor environment.

### Data Management

Data entry is completed separately by each centre, and the data is subsequently cleaned and merged for analysis. A single database has been generated, with the final copy held at one centre only. Data is being analysed on an on-going basis, in particular the analysis of early data to 15 months is well underway.

## Results

The demographics of the cohort are shown in Table 2. A total of 1105 children were recruited. The retention rate at three months was 96.3% and at fifteen months was 91.4%. As of April 2006, a total of 421 children have reached age six years, and have undergone the six-year assessment in Wellington; 264 children have been assessed in Christchurch (which started recruitment later and will finish six-year assessment in 2007). 127 children have to date completed the eight-year questionnaire in Wellington. 15.2% of the children at recruitment have been identified as Maori. A positive family history of asthma, eczema or hay fever has been identified in 84% of children.

**Table 2:** Demographics of the New Zealand Asthma and Allergy Cohort

	**Christchurch**	**Wellington**	**Total**
Total number of children recruited	552	553	1105
Total number with 3 month questionnaire data	535 (96.9%)	529 (95.7%)	1064 (96.3%)
Total number with 15 month questionnaire data	505 (91.4%)	506 (91.5%)	1011 (91.4%)
Male:Female (ratio %)	283:269 (51.3:48.7)	278:275 (50.3:49.7)	561:544 (50.8:49.2)
First born	41.3%	50.5%	45.9%
Second born	37.0%	34.0%	35.5%
Third + born	21.7%	15.6%	18.6%
Number of multiple births	10	2	12
Gestation: Mean (range)	39.6 (33.0–43.0)	39.6 (32.0–43.0)	39.6 (32.0–43.0)
Maternal age at birth: Mean (range)	29.9 (16.3–46.7)	30.3 (15.5–45.6)	30.1 (15.5–46.7)
Percentage Maori Children† *	11.6%	18.9%	15.2%
Percentage with Maori Mother *	5.6%	9.4%	7.5%
Family history† § n = 1046	85.2%	82.9%	84.0%
Smoking during pregnancy †	21.1%	20.8%	21.0%
Household smoking †	33.6%	31.8%	32.7%
Cord blood obtained	91.7%	87.5%	89.6%

† Collected at age 3 months

* Maori has been defined to include both sole Maori and Maori plus other ethnicity, using questions derived from the 1996 New Zealand Census.

§Family history has been defined to include either parent with asthma, hayfever or eczema. Fathers not living with the child were not asked this question

The gender ratio in the cohort is 561:544 (50.8%:49.2%) (Male:Female), with 45.9% being first-born, 35.5% being second-born, and 18.6% being third-born or higher. Maternal age ranges from 15.5 years to 46.7 years.

## Discussion and Conclusion

Cohort studies always present a considerable challenge to recruit and retain a representative sample of a population. We have assembled a population based birth cohort of over 1000 children in Wellington and Christchurch with good retention rates. We specifically sought to obtain a representative population sample rather than to recruit families with a history of atopy, allergic disease or asthma in order to provide greater generalisability of the results. We were reliant on midwives for recruitment of mothers in their care. We randomly sampled midwives from a variety of maternity care facilities including local hospital birthing units, secondary care facilities and a number of independent midwife practices. We met with the midwives regularly and asked them to approach all mothers in their care and to keep a record of all mothers that declined to participate. This proved very difficult in practice and we have no reliable information on how many mothers declined, although we believe that in almost all cases midwives did approach all mothers in their care. We are confident however that the cohort sample is largely representative of the wider New Zealand population, based upon our comparisons with national demographic statistics. For example, in the NZ Census of Populations and Households 2001, 14.7% of the population identified as Maori. Overall, in Christchurch and Wellington, 15.2% of the cohort children have been identified as Maori. Also parity in the cohort is not dissimilar from the NZ National Statistics [34]. We had a slightly higher proportion of 1^st ^born children (46% cf 41% nationally), the same proportion of second born children (36%), and a slightly lower proportion of children with higher sibships (19% cf 24% nationally). The national statistics were based on births within marriages which may explain the difference. Recent birth cohort studies have tended to recruit from University or academic teaching hospitals where high risk pregnancies or deliveries are likely to be over represented and may therefore differ from the general population. The issues raised by non-random sampling for these birth cohorts are discussed by Keil and co-workers who review recent European asthma and allergy birth cohort studies as part of the GA^2^LEN (Global Allergy and Asthma European Network) initiative. [14].

Another concern in cohort assembly is participation bias, the inevitable increased participation of subjects who have a family history of the conditions being studied. This cannot be prevented but the extent to which it is present can be assessed by collecting information on non-responders and those not wishing to take part. In assembling this cohort such information was not available for the reasons discussed above. To assess participation bias we have compared a family history of asthma, eczema or hay fever amongst the cohort infants with the participants in the Dunedin multidisciplinary cohort study [8]. We defined family history as either parent having a history of asthma, eczema or hayfever. Using this definition in the most recent round of the Dunedin study (response rate 96%), when subjects were aged 32 years, (similar to the average age of mothers in our cohort) 70% of female cohort members reported a history of one or more allergic diseases and 64% of males, (personal communication Dr R. Hancox, Deputy Director, Dunedin Multi-disciplinary cohort study) showing that 89% of random pairs of the Dunedin cohort would have a history of allergic disease. This suggests that our cohort is a reasonably unbiased sample with no obvious evidence of participation bias due to a family history of allergic disease.

The initial decision to use the Quintest equipment for the fifteen-month skin test was made after initial piloting suggested difficulties in performing SPT's using standard prick methods in 15 month old infants. It was considered that the Quintest would offer a number of advantages including ease of administration, reducing distress because of quicker testing times; standardisation of technique; less effect from child movement, and the simultaneous completion of all tests. During the course of the study however we became concerned that the Quintests were producing smaller wheals than would have been expected using standard techniques. In 2000, we conducted a study to examine the Quintest and compare it to a standard prick-through application. Not only did we confirm smaller wheal sizes from the Quintest, amongst atopic adults, but we also we showed that this would lead to a 25% misclassification of atopic status, when using a mean wheal diameter of 3 mm as a cut off [35]. The Quintest also tended to produce more positive responses to the negative control. We also found that there were significant differences between two commonly used sources of commercial allergen preparations, with allergens from one source consistently producing smaller wheals regardless of the methods used to apply them. Once again the source of allergens also led to a 25% misclassification of atopy. In order to reduce the impact of the smaller wheal sizes from the Quintest we have used the histamine to wheal ratio to define atopy. Thus for each allergen after subtraction of the negative control, atopy was defined as a histamine allergen ratio ≥0.5. This ratio assessment has been recommended by a number of investigators particularly in young children [22] and reduces the impact of smaller wheal sizes due to the Quintest device. It is important to recognise that the assessment of atopic status in infancy and early childhood can only ever be a partial assessment. Many children will exhibit transient food sensitisation and many others will not yet have developed sensitivity to environmental allergens.

A number of birth cohorts have been established internationally to study a wide variety of factors associated with the development of atopy, allergic disease and asthma [14]. Given the recent hypotheses that the development of these conditions involves a complex interaction between genetic factors, and timing and magnitude of different environmental factors, there are significant advantages to investigators sharing testing protocols and information at early stages, in order to test hypotheses under different environmental conditions. The GA(2)LEN project funded by the European Union is an example of this approach. The high prevalence of allergic disease and asthma in New Zealand, with high allergen levels and low industrial pollution levels, allows testing of hypotheses generated by both cross-sectional and longitudinal studies in a unique environment. We present information about our cohort to contribute to this process and encourage interested researchers to contact the corresponding author for further information.

## Competing interests

The author(s) declare that they have no competing interests.

## Authors' contributions

ME is the principal investigator for the Christchurch arm of the study, and has been involved in the design of the study and the investigations, acquisition of data, and analysis and interpretation of data.

GIT was lead principal investigator for the study until 2004, and has been involved in the conception and design of the study and the investigations, acquisition of data, and analysis and interpretation of data.

TI has been involved in the design of the study and the investigations, acquisition of data, and analysis and interpretation of data.

KW has been involved in the design of the study and the investigations, acquisition of data, and analysis and interpretation of data.

DF has been involved in the initial design and conception of the study.

JC is currently lead principal investigator for the study and has been involved in the conception and design of the study and the investigations, acquisition of data, and analysis and interpretation of data.

All authors have read and approved the final manuscript.

## Pre-publication history

The pre-publication history for this paper can be accessed here:


